# “*ANZANSI Program Taught Me Many Things in Life*”: Families’ Experiences with a Combination Intervention to Prevent Adolescent Girls’ Unaccompanied Migration for Labor

**DOI:** 10.3390/ijerph192013168

**Published:** 2022-10-13

**Authors:** Ozge Sensoy Bahar, Alice Boateng, Portia B. Nartey, Abdallah Ibrahim, Kingsley Kumbelim, Proscovia Nabunya, Fred M. Ssewamala, Mary M. McKay

**Affiliations:** 1 Brown School, Washington University in St. Louis, Campus Box 1196, One Brookings Drive, St. Louis, MO 63130, USA; portianartey@wustl.edu (P.B.N.); nabunyap@wustl.edu (P.N.); fms1@wustl.edu (F.M.S.); 2 Department of Social Work, University of Ghana, Accra P.O. Box LG419, Ghana; aboateng@ug.edu.gh; 3 School of Public Health, University of Ghana, Accra P.O. Box LG419, Ghana; ibdallah4@gmail.com; 4 BasicNeeds Ghana, Tamale P.O. Box TL1140, Ghana; kingsley.kumbelim@basicneedsghana.org; 5 Vice Provost Office, Washington University in St. Louis, One Brookings Drive, St. Louis, MO 63130, USA; mary.mckay@wustl.edu

**Keywords:** intervention acceptability, evidence-based intervention, child labor, unaccompanied migration, adolescent girls, qualitative, Ghana

## Abstract

Approximately 160 million children work as child laborers globally, 39% of whom are female. Ghana is one of the countries with the highest rates of child labor. Child labor has serious health, mental health, and educational consequences, and those who migrate independently for child labor are even at higher risk. Yet, evidence-based efforts to prevent unaccompanied child migration are limited. In this study, we examined the acceptability of a family-level intervention, called ANZANSI (resilience in local language) combining two evidence-based interventions, a family economic empowerment intervention and a multiple family group family strengthening intervention, to reduce the risk factors associated with the independent migration of adolescent girls from the Northern region to big cities in Ghana. We conducted semi-structured interviews separately with 20 adolescent girls and their caregivers who participated in ANZANSI. Interviews were conducted in the local language and transcribed and translated verbatim. Informed by the theoretical framework of acceptability, the data were analyzed using thematic analysis. The results showed high intervention acceptability among both adolescent girls and their caregivers, including low burden, positive affective attitude, high perceived effectiveness, low opportunity costs, and high self-efficacy. The study findings underline the high need for such interventions in low-resource contexts in Ghana and provide the foundation for testing this intervention in a larger randomized trial.

## 1. Introduction

Approximately 160 million children (ages 5–17) across the globe work as child laborers, 39.3% of whom are female [1]. In addition, 35% of child laborers do not attend school [1]. Sub-Saharan Africa (SSA) has the highest rates of child labor (23.9%) in the world [1], with Ghana being one of the countries with the highest rates of child labor for children ages 5–14 (13%) in the region, including harvesting, cocoa production, and fishing [2,3]. Child labor is a significant public health concern due to serious health, mental health, and educational consequences for children [4,5,6]. Poverty is a major contributing factor to unaccompanied rural-urban child migration and drives the high incidence of child labor across SSA [7]. 

Child labor constitutes a significant threat to children’s education, undermining their chance to break the cycle of poverty [6,8,9]. This concern is even more critical for female children and adolescents as education has benefits specific to girls, including delay in marriage age, higher chances of entering the formal labor market, and better health outcomes for them and their children when they become mothers [10]. Relatedly, while engaging in child labor can be a critical household survival strategy, its negative consequences make it a dubious investment in the long run [11,12].

Statistics in Ghana show that girls are more likely to engage in hazardous child labor than boys [13], which is defined as “*work which, by its nature or the circumstances in which it is carried out, is likely to harm the health, safety or morals of children*” in Article 3(d) of International Labor Organization Convention 182 [1]. Girls’ education is often compromised due to limited financial resources and traditional gender norms [14,15]. As a result, adolescent girls living in poverty increasingly make up the majority of the North–South migrants in Ghana, working as head-load carriers, referred to as “Kayayei” [12,13]. Carrying head loads allows girls to immediately earn small amounts of money without any capital required [16].

In addition to poverty, kayayei girls identify family factors as reasons for their unaccompanied migration to big cities for work [14,17,18]. Families, including high-quality caregiver-child relationships, play a critical role in healthy adolescent development and should be included in interventions targeting adolescents [19,20,21,22]. Studies have identified child maltreatment as a risk factor associated with unaccompanied child migration [23,24]. Moreover, parental gender norms and attitudes toward education are associated with parental decisions on child labor [25]. Hence, strengthening family supportive processes and attitudes is critical.

While kayayei girls are identified as a high-risk population, preventive efforts are limited [2]. Existing interventions often address only one aspect of the problem without targeting family needs/factors that lead to unaccompanied migration. The multiple risk factors, primarily poverty and family factors, leading to the unaccompanied migration of children and adolescents for work necessitates a comprehensive approach [26], including combination interventions—a combination of two or more interventions—designed to address these risk factors simultaneously.

Additionally, while there are studies testing the effectiveness of interventions targeting youth in low and middle-income countries, intervention acceptability studies among adolescents in LMICs, specifically in Africa, are still limited [27]. Acceptability is necessary—though not sufficient by itself—for intervention effectiveness [28]. From the participant’s perspective, the content, context, and quality of care may contribute to acceptability [28]. Intervention acceptability studies are critical because interventions have higher uptake and effectiveness if adolescents find them acceptable [28,29]. A recent systematic review of peer-reviewed studies (published between January 2010 and June 2020) assessing intervention acceptability with youth (aged 10–24) in Africa mainly included studies focused on HIV or sexual and reproductive health outcomes [27]. Moreover, these interventions were either single interventions or only explored the acceptability of select components of a combination intervention, leaving out the opportunity to assess the acceptability of an intervention as a package.

Hence, in this study, we explored the acceptability of the ANZANSI family program (hereafter ANZANSI, resilience in Dagbani, the primary local language in the Northern region of Ghana) among adolescent girls and their caregivers. The ANZANSI program focuses on adolescent girls at risk of dropping out of school by combining two evidence-informed interventions tested in SSA: family-level economic empowerment intervention to create and strengthen household financial stability and multiple family group intervention to address beliefs around girls’ education, gender norms, and child labor as well as family functioning. The overall goal of the intervention is to reduce the risk of school dropout and unaccompanied migration for child labor among adolescent girls in Northern Ghana.

## 2. Methods

### 2.1. Overall Study

Informed by asset theory [30,31] and parental ethnotheories framework [32], this pilot study is a clustered randomized clinical trial (R21HD099508) designed to test the feasibility, acceptability, and preliminary impact of the ANZANSI combination intervention (family economic empowerment + family strengthening) on child and caregiver outcomes (see study protocol [33]). One hundred adolescent girls and their caregivers from 10 schools in northern Ghana were enrolled in the study. The 10 schools were randomly selected from a list of medium size schools in the study area with the highest rates of female student dropout and at least at 500 m distance from each other. The randomization to treatment versus control arms was conducted at the school level (n = 50 dyads in each arm; 10 per school) to avoid contamination. The inclusion criteria for adolescent girls were: (1) enrolled in school and living within a family (defined broadly to include extended and non-kin family), (2) ages 11 to 14, (3) skipping school in the past academic term (with at least 10% of unexcused absences) and, (4) capable of giving consent. The caregiver inclusion criteria were: (1) self-identified as the primary caregiver of the adolescent girl and, (2) capable of providing informed consent (see Sensoy Bahar et al. 2020 for more details on study design) [33].

**Intervention Description (ANZANSI Family Program).** The ANZANSI intervention combined two evidence-based interventions tested in the SSA: Family economic empowerment intervention to address household financial stability and multiple family group (MFG) family strengthening intervention to address family relations and norms. All participants received transport refund and refreshments for each session they attended.

***Family Economic Empowerment Component.*** The family economic empowerment (EE) intervention has been tested in Uganda, with positive health, mental health, and academic outcomes and material well-being for adolescents impacted by poverty and HIV [21,34,35,36]. The intervention consisted of: (1) financial literacy workshops, (2) child development account (CDA) and, (3) family income-generating/microenterprise promotion component.

1. ***Financial Literacy Workshops:*** This component included four 1 to 2 h sessions, each session covering the following, respectively: (i) financial literacy and budgeting (money management and setting financial goals and the importance of budgeting); (ii) asset-building and the means through which asset-building occurs; (iii) bank services (advantages of using banks and how banks work including how to complete deposit and withdrawal forms); (iv) debt management (borrowing money, good versus bad loans, cost of borrowing). The workshops were facilitated by staff from BIBIR Ghana, one of the two local implementation partners in Ghana.
2. ***Child Development Accounts (CDA).*** The study partnered with Agricultural Development Bank, a local public financial institution, and opened savings accounts known as Child Development Accounts (CDA) for all intervention group participants in the name of the adolescent girl with her caregiver as a co-signer. Any of the participant’s family members, relatives, or friends were allowed and encouraged to make deposits into the CDAs. The study then matched the account at a match rate of 2:1 with money from the ANZANSI program. Every equivalent of USD 1 the participant and her family saved (in Ghana currency) was matched by USD 2 from the study project. The matching cap (the maximum amount of family contribution to be matched by the intervention program) was set at an equivalent of USD10 per month per family or USD90 for the 9-month intervention period. Thus, adolescent girls who saved the maximum amount had USD 270 at the end of the intervention (USD 90 in their savings plus USD 180 from the match). This money was dedicated to the participant’s schooling and/or to starting a small family business. Participants were only allowed to withdraw their own money (but not the matching funds) only in case of emergency during the intervention. Participants could only access their CDA after completing the intervention.
3. ***Income-generating activities (IGA).*** Following the completion of financial literacy workshops, participants were trained on income-generating activities in two 1-to-2 h sessions. The IGA training workshops introduced participants to the concept of IGA and microenterprise. Participants were guided to choose an IGA of their choice and based on their choice, facilitators assigned technical staff to offer financial advice and guidance to participants on establishing and managing their chosen IGAs. As a means of knowledge sharing, participants shared with fellow participants their IGA experiences, including project overview, lessons learned, economic and social gains, challenges, and recommendations.

***MFG Family Strengthening Component***. Informed by the family systems, structural family, and social learning theories [22,37], this intervention, originally developed for children in low-resource communities in the United States, is an evidence-based multiple family group intervention for children and adolescents whose families struggle with poverty and its associated stressors [22]. The manualized 16-session intervention is organized around the skills and family processes referred to as the 4Rs (Rules, Responsibility, Relationships, and Respectful Communication) and 2Ss (Stress and Social Support) [22]. The sessions offer content, skill-building and practice activities to foster learning and interaction among members of a family as well as with other families in the sessions [22]. During the sessions, children and caregivers may complete activities together or split to reconvene later for sharing and discussing as a larger group. This intervention has been found effective in improving child and caregiver outcomes in Uganda [34,38].

**Figure 1:**
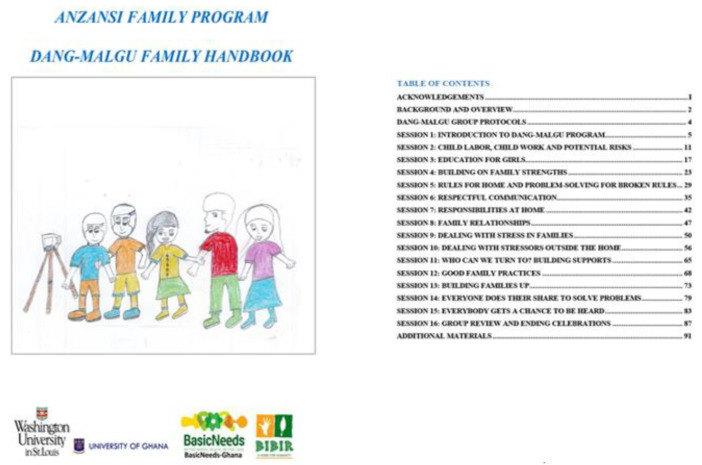
Dang-Malgu family handbook.

For this study, we further tailored the intervention that was already adapted (named Dang-Malgu, Family Togetherness in Dagbani, the local language in the study area) and tested in Ghana as part of another NIH-funded study (SMART Africa Center, U19MH110001) [39,40]. Specifically, sessions 2 and 3 were revised to focus on child labor, child work and associated risks; and education for girls, respectively (see Figure 1).

Each multiple family group (one per treatment school) involved 7 to 10 families, with at least two generations of a family present in each session (the adolescent girl and an adult caregiver). Sessions were facilitated by two school health education program (SHEP) coordinators at each school. SHEPs were trained by staff already trained in MFG at BasicNeeds Ghana, the implementation partner. Each session lasted between 60 to 90 min and was delivered weekly in schools during the weekends.

### 2.2. Sample Selection and Characteristics

Twenty dyads (20 adolescent girls and 20 caregivers) from the treatment arm (four dyads per treatment school) were randomly selected and invited to participate in semi-structured qualitative interviews. Adolescent participants’ age ranged between 12 and 14 (mean = 13.85 years). Eighty-five percent of them were in the first year and 15% in the second year of Junior High School. Eight percent reported that both their parents were still alive and 70% reported their biological parent as their primary caregiver. The median number of people in the household was 16, and the median number of children was six (see Table 1).

The age range for caregiver participants was between 29 and 80, with an average of 42.85. In total, 75% of the caregivers reported being engaged in informal employment and 15% in formal employment. A total of 5% reported being unemployed, and the remaining 5% had retired (see Table 2).

### 2.3. Data Collection

Face-to-face semi-structured interviews were conducted with adolescent girls and their caregivers separately following the completion of the ANZANSI intervention, between December 2021 and January 2022. The interviews focused on participants’ (1) experiences with the intervention, including each of its specific components; and (2) key multi-level (individual, family, contextual, and program) influences affecting program participation. The interview questions included: “Could you please tell us a little bit about why you decided to participate in the ANZANSI program?” “Overall, what did you think of what was covered in the sessions?” “Can you tell us about your experience attending the sessions?” “What information or skills covered in the sessions have been most helpful to you?”, “What were the things/factors that made it easy for you to attend the sessions?” “What were the factors that made it difficult for you to attend the sessions? How did you overcome those barriers?”.

The interviews were conducted in Dagbani, the widely spoken language in the study region. The interview guide was translated (English to Dagbani) by a professional translator and reviewed by study team members fluent in both languages to ensure they sounded natural and conversational, and conveyed the same meanings intended. The interviews were conducted by research assistants fluent in both Dagbani and English and trained extensively by the in-country PI with qualitative research expertise. Lasting between 60 to 90 min (mean = 75 min), each interview was conducted in a private place with only the research assistant and the participant present. All interviews were audio-recorded. To maintain participant privacy and confidentiality, pseudonyms were used in the presentation of study results.

### 2.4. Theoretical Framework Guiding the Data Analysis

We used Sekhon et al.’s [28] theoretical framework of acceptability (TFA) to guide our analysis. According to TFA, acceptability is “a multi-faceted construct that reflects the extent to which people delivering or receiving a healthcare intervention consider it to be appropriate, based on anticipated or experienced cognitive and emotional responses to the intervention” (Sekhon et al., 2017, p. 4) [28]. Specifically, TFA consists of seven constructs, namely affective attitude, burden, perceived effectiveness, ethicality, intervention coherence, opportunity costs, and self-efficacy [28]. Affective attitude refers to how an individual feels about the intervention, prior to (anticipated) and after taking part (experienced) in the intervention. Burden includes the perceived amount of effort that is required to participate in the intervention (anticipated) and the amount of effort that was required to participate (experienced). Perceived effectiveness also includes both the extent to which the intervention is perceived to be likely to achieve (anticipated) and to have achieved (experienced) its purpose. Ethicality is defined as the extent to which the intervention aligns with the value system of the participating individual. Intervention coherence refers to the extent to which the participant understands the intervention. Self-efficacy is the participant’s self-confidence that they can perform the behaviors that are required for intervention participation. Finally, opportunity cost also includes both the extent to which benefits, profits, or values one must give up engaging in the intervention (anticipated); and the extent to which one gave up when engaged (experienced).

### 2.5. Data Analysis

All interviews were transcribed verbatim and translated by a professional translator. The transcripts were reviewed by the research team to have a broad understanding of the content and to identify topics for discussion/observation. Analytic induction techniques were used for coding, utilizing both sensitizing concepts and identifying emergent themes (open coding) [41]. The themes were broken down into smaller, more specific units until no further subcategory was necessary. The transcripts were coded by two research team members independently, and disagreements were resolved through team discussions. Subsequent analyses compared themes across participants, and between caregivers and adolescent girls for similarities, differences, and relationships. Peer debriefing was used for rigor, where the codes and corresponding excerpts were presented to two members of the research team who were not involved in the data analysis to discuss the plausibility of the themes [42].

## 3. Results

To fully capture the acceptability and appropriateness of the ANZANSI intervention for the participating adolescent girls and their caregivers, we explored their motivations and concerns about enrolling in the program as well as the barriers and facilitators to their attendance. We also inquired into their thoughts regarding the different characteristics of the intervention, including content relevance, group format, day and time of the sessions, and session facilitators (see Table 3).

### 3.1. Motivation to Participate in the Program

As part of their overall experience, the interviews explored what motivated the adolescent girls and their caregivers to enroll and what their initial concerns -if any- were about the program. Caregivers’ motivations reflected several aspects of the program. Some caregivers emphasized the economic empowerment component as of interest to them. For instance, both Nabila and Nadia were motivated by the fact that the program would provide training on generating income, saving, and business management.

***Nadia:***  *When they said they were going to train us on how to manage our business effectively and also how to generate income, to be able we save part of our earnings. Those factors motivated me to join the program.*

Samira and Zaina were interested in the economic empowerment aspect in relation to their daughters’ education. By learning more about income-generating activities and saving, Zaina hoped that she would be able to save so that she could keep her daughter in school.

***Zaina:***  *I was joining it with an intention, for instance concerning my child’s education; I keep thinking over it a lot. And someone decided to come to my aid about it. Concerning the income-generating activities they taught us, that was why I paid attention to it because I heard all the things that I have never heard in the program. I intended to learn about business strategies and my child’s education; how I will save money so she will not drop out of school because some children have dropped out of school because of lack of finances.*

Taufiq appreciated the program’s focus on girls and education. As a father to a girl child, he was motivated to learn more about *“girl-child education and what we could do to promote their wellbeing and also, how they would be guided to stay away from bad peer influence.”* He went on to add that he appreciated the preventive approach of the program that would help girls remain in school and be *“well-educated”*.

***Taufiq:***  *The reason I joined is that my child is a girl. And you people have chosen the girls to help them to prevent them from going to ‘kayaye’ for them to be able to be well educated for the future wellbeing of them and us. And I have seen that it is a good course. So that is why I joined it.*

Learning about *“managing family issues, especially how we can properly handle our children’s affairs”,* as expressed by Bushira, a female caregiver, was a motivator for many caregivers. Waramatu wanted to learn about how to take care of her children in all aspects of their lives, including education.

***Waramatu:***  *What I aimed at learning was for instance the kind of skills they were teaching us: this is how you are supposed to take care of your child. Concerning the school, you should be involved in the education. This is how they should be living. Whenever they come home from school, this is what they should be doing. This is how you should treat them. Actually, at the time I was joining it, it is what they were telling us about child upbringing that made me join. It is that aspect that thrilled me.*

For adolescent girls, one of the most cited motivators was the focus on family and the opportunity to improve family relations. For instance, Ayat was interested in learning about “*how to respect and promote family*”. In fact, respecting parents came up frequently. Ameeda was motivated to learn about “*child responsibility and respect for our parents*”. Hajara mentioned the things the program taught her about respecting her caregivers when asked about what she hoped to learn from the program.

***Hajara:***  *I learned things like having respect for your parents and listening to them very well when they speak to you and not insulting them; then also respecting people who are older than you. And they also taught us things that can make you sit with your parents to have a conversation.*

Another motivation for adolescent girls was the focus on the “girl child” and her education. Suweba believed that the program would help her stay in school and further her education instead of migrating to become “Kayayei”.

***Suweba:***  *I want it to help me in terms of my education or the person I will grow up to be. You said you were going to help us so that we will not go to become Kayayei (load carriers) and also help us further our education, and we will not even think of going to places that will not be beneficial to us.*

Along similar lines, Jamila and Waseela were motivated by the emphasis on girls’ education. Jamila hoped to learn strategies to cope with the challenges that may get in the way of her education.

***Jamila:***  *I was also expecting to learn strategies that can help to cope with educational challenges. The aspect of the program that motivated me much has to do with the mission and vision of the program: how it encourages parents to send their children to school.*

For some, it was a combination of different aspects of the program. For instance, Patricia cited financial literacy and the opportunity for a better future as the reasons why she joined the program. For Felicia, the program was of interest because she wanted to remain focused and learn about respect and savings.

***Felicia:***  *I don’t want to be roaming aimlessly or following after men that is why I joined the program. What was appealing to me is when you said you were going to teach us how to respect [our family] and how to save in the bank.*

*Initial concerns about program participation*

We also asked adolescent girls and their caregivers what made them hesitate to participate when they first heard about the ANZANSI program. Most of the caregivers said that they had no concerns, as illustrated by Jumai, a female caregiver who was eager to join the sessions: *“There was nothing preventing us from joining. Whenever there was a meeting, I was ready to go.”* Similarly, Karim weighed the pros and cons of participating in this program as opposed to his other responsibilities and decided that the benefits were worth investing time in.

***Karim:***  *When you are faced with two issues, you look at the most important one because I found that the benefit I was gaining here, I realized that even if I left everything to take part in meeting it would be useful for me. Because they taught me how to take care of my child, my family, and so on. That is why I left everything just to participate in the program.*

A few caregivers were initially worried about the potential time commitment and that they would not have time for their business. For instance, Taufiq was concerned that the program would take time away from his trading activities and hinder his income. However, he eventually realized that attending the program did not cost him much.

***Taufiq:***  *Actually, my fear was about the time it was consuming. And I was afraid that it will take all the time that I was supposed to spend on my trading activities from where I get my little earnings to feed myself and my family. However, I just pursued it with great courage. And indeed, when I joined, it has not cost me much.*

Zakiya mentioned that while the timing of the sessions did not initially work for her, she adjusted her schedule to be able to attend. Similarly, Waramatu expressed time commitment as an initial challenge, which she accommodated by adjusting her schedule.

***Waramatu:***  *Whenever there is something to prevent me from attending the program, I try to do all that I can and put aside that thing and attend the program. After I close from the program, then I will come back and do that work.*

Similarly, almost none of the adolescent girls had any initial concerns about the program. Two participants, Sherifa and Leila, mentioned that some people in the community said they should not trust the program, but they decided to participate anyway, as illustrated by Leila, *“Some people said this project was a lie but we had confidence in the project and followed it till now and we are benefiting from it”*.

### 3.2. Facilitators to Program Attendance

The attendance rates, including late arrival, for the 4 FLT and 16 MFG sessions were 98% and 99%, respectively, for the 47 dyads who participated in the intervention. The attendance for the two IGA sessions was 88%, lower than other components due to one of the sessions being scheduled on a heavy rain day. Hence, the overall attendance rate across sessions was 95 percent. Families were allowed in the sessions until 10 min after the session started. However, there were instances where more flexibility was needed and sessions started 10–15 min later than the usual start time, especially during the rainy season. Both the caregiver (or their delegate) and the adolescent girl had to be present to participate in the sessions.

For many caregivers in our qualitative sample, the relevance of the session content to their families was what motivated them to attend the sessions. Abiba noted that the benefits of attending outweighed everything else.

***Abiba:***  *I was looking at the benefits I will gain from it because what we were learning gave me some motivation and courage, and also made me learn things I do not know. Even if I had something challenging me [to attend], I would come for the training and later go to solve the problem.*

Karim shared similar thoughts that what made it easy for him to attend *“was the information they were giving us because they taught us how to make money and to save money without spending it”* and that he did not miss any session.

For a couple of caregivers, another facilitator was that the sessions were delivered on the weekend, which did not interfere with the caregivers’ work. For instance, Samira mentioned that *“because the training was always scheduled on Saturdays, I used to come and after the training, I returned to my daily activities”*.

Some caregivers mentioned the transport refund provided at the end of the session as a facilitator. Given that the program was implemented in resource-poor communities, covering the cost of weekly transportation would have been a financial burden on the families. Zaina spoke about how it would have been hard for her to attend the sessions without the reimbursement.

***Zaina:***  *What made it easy for me is the transportation money they used to give us whenever we ended the meetings because from my village to this place the fare is 10.00ghc. So, if the transportation money was not part, that would have made me not be able to come even when I didn’t intend to miss it. But because there was transportation money, if you are just ready and you come out, you will attend the meeting.*

She went on to add that her husband always encouraged her to attend the sessions. He became a firm believer in the program as Zaina shared with him what she learned at the end of each session.

***Zaina:***  *Whenever we just attend these sessions and I go home, I will tell my husband what happened. This is what they said, this is what they said. I will also explain this to him. So because of that, whenever we have work to do and they call, he will say that you should leave work and come and listen to what they have for you.*

Adolescent girls also mentioned the content of the sessions as one of the reasons that motivated them to come to the sessions. For instance, when asked about what made it easy for her to attend the sessions, Ayat said, “*They were teaching us how you will save money, how to fill the deposit slip, and what you supposed to do when you go to a bank…. They were educating us on how a child should live.*” She added that she experienced no challenges in attending the sessions. Similarly, Patricia who never missed a session shared, “*We never missed a session because we know it is something that is going to help us in relation to our family and education*”.

### 3.3. Barriers to Program Attendance

Some caregivers mentioned that other family responsibilities or sickness interfered with their attendance at times. However, even in those instances, caregivers attempted to make sure that another adult family member attended the session in their place, as illustrated by Taufiq, “*From the beginning till the end, I was only absent once which I told my wife to represent me, and this absenteeism was a result of a journey I could not avoid*”.

Though most adolescents did not identify any challenges to attendance, among the few who did, transportation was a common theme, especially finding a taxi. Sherifa mentioned that it was not easy to find taxis and one had to stand in line for a long time before getting a ride. Jamila had a similar experience. While she made sure she completed all her other responsibilities before the session, she also reported struggling with finding a taxi though that did not prevent her and her caregiver from attending all the sessions.

***Jamila:***  *Because we were aware of the day of the training, we usually prepared accurately by performing other obligatory duties before the meeting day. Getting a taxi was my challenge, they always leave early in the morning, and they do not usually return till they get passengers.*

Some adolescents expressed other commitments, such as travel and housework that made it challenging to attend the sessions. For instance, Halima shared that she had housework before attending the sessions, which made her come late to some sessions. Hajara had housework, work for income, and an Arabic class that she stopped going to so that she could attend ANZANSI sessions.

***Hajara:***  *The challenge was that there were a lot of tasks on me in the house. And if I also leave it and attend the program, I will still have to go back home to do it. And after finishing the work in the house, I will still have to go and work to get money to feed myself. And I did my best to do the work well to be able to overcome it.*

### 3.4. Content Relevance and Perceived Impact

The participating families found the content of the different components of ANZANSI highly relevant to their needs. Karim shared everything he learned in the program, touching upon all its components.

***Karim:***  *They taught us how to trade, save, what to do to be healthier, and how to take care of your family, your household, and your friends. How to relate with people in the community, I learned all these through ANZANSI.*

Most caregivers noted that they did not have a bank account prior to the program and did not know how to open one until they participated in the intervention, as expressed by Saudatu, “*I wanted to have a bank account, but I didn’t know what to do. It was through this program that I had the opportunity to open one and I have benefited a lot in the program*”.

Asiya shared that it was through ANZANSI that she learned how to save money at the bank. The matched saving was appreciated by the caregivers. Yet, it was also a challenge given the limited resources as illustrated by Waramatu, “*Actually, the challenge [to save money] was that sometimes we may not be able to even get what we are supposed to survive on. And so, when the day comes, it would be a challenge for me*”.

Even when it was not much, caregivers made every effort to save. For instance, a male caregiver, Isaac, made sure to deposit at least some of the transport refund provided at the end of each session, especially given the unpredictable nature of farming.

***Isaac:***  *The transportation refund that you gave us helped me… I used the transport refund to open the account. Every month I go to save 50 Ghana cedis and continue to do my work again. We farmers, sometimes it would take days and you would not work. But with the accounts, I always made sure to get the 50 Ghana cedis to save at the end of the month.*

Zaina, who did not know how to open an account before the intervention, made every effort to deposit during the program. She continued to do so even after the matching ended. She shared that as a result of the savings, she did not have any concerns about paying the school-related expenses for when her daughter resumed school.

***Zaina:***  *I didn’t know how I can open an account. So, we are very happy about it. The payments have finished but we still have not stopped depositing money into it. We keep doing it. We will be selling and saving little by little. Every day, you will save 2ghc, by the time the day is due, it will be up to the target amount and then you will go and deposit it into the account…… When they resume school, I no longer have any problem regarding how we will be buying her school stuff.*

Saudatu did not think that *“poor people”* could save in the bank until she joined the program. Since then, she deposited even the little money she had.

***Saudatu:***  *I have put the knowledge that I acquired into practice in opening a savings account in the bank. I didn’t know poor people could do savings in the bank and now that I know, even if I have pesewas (coins) I can deposit them in the bank.*

Isaac discussed how bank account opening and financial literacy helped both his family and extended kin.

***Isaac:***  *The skills I have gained with regards to the opening of bank accounts and the financial literacy aspect have helped my family. Now if we have a family financial issue, I will be the one they will contact to resolve the issue. So, it has helped all of us.*

Another caregiver, Hadiza, shared what she learned in financial literacy training with her mother and sister. She added that she was able to expand her business using the knowledge she acquired in the financial literacy and income-generating sessions.

***Hadiza:***  *I taught my mother and my sister about the skills in financial literacy training, which enlightened me about my business management.… I was able to expand my business through the knowledge I acquired in the training. The knowledge of savings and banking activities helped me much, and it enlightened me about the need to engage in savings.*

Caregivers also commented on the relevance of the family strengthening sessions to their families and proceeded to expand on how their families benefited. Waramatu shared that the skills and knowledge she learned in the sessions helped her to deal effectively with her children who “*misbehaved*”, which she found satisfying.

***Waramatu:***  *Yes, I used it to work. And honestly, there was satisfaction because after acquiring this information and getting home, when a child was misbehaving, and then I talked to her, I realized that she would also listen to me, and behaved well.*

For some caregivers, like Jumai, the family strengthening sessions helped the family come together and feel united, something she thought was missing in her family, *“There was no unity in the family. Everyone was doing their own things. But since we started this thing, we are now united in the family and there is peace in the family”*.

Caregivers also mentioned that they learned how to better relate with their children. For Zaina, she learned that she could sit and have a conversation with her children –something she thought was not possible before.

***Zaina:***  *We didn’t know that it is important for you and your child to sit together and have a conversation. So, we got all those skills in ANZANSI. There are certain issues that when you are sitting with the child, you can be asking her about them, and she will tell you. But we, as members of the Dagbamba tribe, we had it that you cannot be sitting at a place and your child too will be sitting there. And it is here that we received all those skills, and we have not stopped using them.*

She went on to add that this was “*really a program for family wellbeing because each and everyone, all of us who were sitting here, we all had each other’s mobile contacts, calling one another. So that is the family we became*”.

For Karim, it was learning about adjusting his expectations from his children in terms of what work they can and cannot do given their capacity. Through the sessions, Asiya realized that being harsh with her children was not the best solution and that she should instead have a conversation with them.

***Asiya:***  *I didn’t know that it is not good to be harsh on your child, you don’t have to make your child fear you. Always make sure you converse with your child or when you are to talk to your child, you should not shout at her. If that continues and she has a problem she can’t share that with you.*

For some caregivers, the focus on girls’ education was important. For Nabila, content covered about girls’ education shifted her perspective on their educational needs, “*Now, I showed more support for the child’s educational needs, as compared to before [the program] where I used to perceive some of her educational needs to be unnecessary*.” As a result of the program, Jumai decided to continue with her daughter’s education and use their savings to pay for school expenses: “*Our plan is for her to progress with her education. When she goes further, we can use the savings together with the help you are offering us to pay her fees*”.

Adolescent girls also raised similar points regarding content relevance and perceived impact. Many of them discussed how both the economic empowerment and family strengthening components were beneficial to them and their families. For instance, Fawzia shared how she was able to save and the potential of expanding their business. She also spoke about how her family was more *“united”* because of the family strengthening sessions.

***Fawzia:***  *We thought that with the savings we have made and the money you added, when we withdraw it, we can expand our business. I used to do little savings at home and when it’s reasonable then I will go and deposit it in the bank. It was great…. [The family strengthening component] has helped to unite my family. They used not to sit together, even they will drive me away anytime I wanted to sit with them but now we can sit together.*

Patricia echoed similar thoughts. She explained how the program taught them they could start saving in small amounts and eventually have enough to start their business. She also discussed how she learned to be respectful towards her elderly and how to *“build peace”* in the family and community.

***Patricia:***  *They taught us that if you have small money already, don’t spend it. Save it in your bank account or mobile money and you keep contributing little by little so that it if gets to the amount that you want to use for the business you remove the money and start the business. The program has helped my family because they used to struggle for money but since they started savings, it is better….The program taught me how to be respectful in life like respecting the elderly so that you will also be respected; and how you’ll build peace in the household and the community.*

Nura shared how she and her family did not know about bank accounts and savings. She also underlined that the main benefit for her was the emphasis on the importance of educating girls. As a result of the program, she saw that her grandmother paid more attention to her education and educational expenses.

***Nura:***  *We didn’t know how to go to a bank to save money or get money and then manage your spending. We didn’t know all this until they came and taught us this….As for me the key benefit of this program was that some people think that it is not compulsory to educate the girl child. But this program made us know that it is good to educate the girl child. I have seen changes in my grandmother. At first, you will come from school and ask for your exam fees, and she will tell you that she doesn’t have money. Now that this program educated her about the benefits of girl child education, when I need something for school, even if she doesn’t have the money, she will give you the day that she will be able to provide it for you.*

Both Felicia and Leila discussed how they benefited from the economic empowerment component. Felicia shared how she learned to make a profit.

***Felicia:***  *I used [the knowledge] in my trading activities, buying with a little amount. For example, I’ll buy a toffee at 0.10p and sell it at 0.20p and go and buy two at 0.20p and sell it at 0.20p each to make an extra 0.20p as a profit.*

In addition to learning how to save, Leila talked about how her family benefited from the family strengthening component as her family was able to come together and feel more *“united”*.

***Leila:***  *My difficulty was how can I save money or where can save my money but after the ANZANSI project came we now know how to save money. They educated us that when you engage in a trade and the little profit you make, you should be able to save some continuously and by that you will be able to gather money. Dang-Malgu [family strengthening component] has helped my family members a lot because there was no unity among most of them but through what I learned here, I was able to share with them and they are now united.*

Hamamat said that her family found going back to the bank *“stressful and difficult”* and did not have any experience with depositing money until they joined ANZANSI. She also shared that through the family strengthening component, she was no longer feeling nervous when speaking to her family members.

***Hamamat:***  *We thought that going to the bank was stressful and difficult because we had never gone to the bank. We used not to deposit money in the bank because we didn’t know how to do that. After we joined it, we have now seen how simple it is to do anything in the bank. And now know the banking rules as well. Thanks to Dang-Malgu, now, when you want to tell your family something or when you have any challenge, you know how you will address it with them. You won’t be nervous or feel intimidated anymore.*

## 4. Discussion

In this study, we explored the acceptability of the ANZANSI program, a family-level combination intervention seeking to prevent the unaccompanied migration of adolescent girls in Northern Ghana for work in big cities, among adolescent girls and their caregivers. We qualitatively explored their expectations and concerns that contributed to their decisions to participate in the overall program, facilitators, and barriers to their participation, as well as thoughts on content relevance and perceived impact, all aspects relevant to program acceptability.

Both the caregivers and adolescent girls were motivated to participate in the ANZANSI and highlighted the different aspects of the program as motivating factors. For caregivers, the economic empowerment aspect was frequently mentioned. Caregivers were excited about the opportunity to open bank accounts and learn about savings, financial literacy, and income-generating activities. Another frequently mentioned motivator was the potential of this program for improving family relations. Only one caregiver mentioned the focus on girls and their education as a motivation. On the contrary, this focus was more frequently mentioned as a motivation among adolescent girls, suggesting the need among this population for more gender-specific programming addressing their unique needs. Adolescent girls also tended to mention the family strengthening aspect more frequently than the economic empowerment aspect of the program as a motivation. When mentioned, economic empowerment was discussed in combination with family strengthening. This difference may be a function of financial stability being more of an immediate concern for adults responsible for their household finances. Regardless of the differences, the motivation for both adolescents and caregivers was to acquire more knowledge that applies to multiple aspects of their lives, which underscores the need for such programs in these communities. In addition, the high motivation of the families to participate in the intervention point to a positive anticipated affective attitude, one of the acceptability dimensions of TFA [28].

Most caregivers and adolescent girls had no initial concerns about participating in the program. The concern expressed by a few caregivers was the time commitment that the program required. While this is a common concern in interventions that require an extended period of time for participation [43,44,45], the fact that only a couple of caregivers mentioned it may speak to the high need and relevance of programs such as ANZANSI among this population. There was no initial hesitation among adolescent girls, even among those who heard rumors that they should not trust the program. Community distrust or skepticism has been widely discussed in the context of health and mental health systems [46,47,48]. The “rumors” in the communities may have resulted from past negative experiences with other service providers. It may also stem from the lack of available programs in the area, which leads to community members questioning its authenticity when a program is offered. While this misinformation did not prevent families from participating, it is a key point to be considered and adequately addressed when implementing programs. Overall, the level of anticipated burden [28] expressed by both caregivers and adolescents was low. In cases where the burden was anticipated and caregivers expected to give up on other profits (e.g., work) -referred to as anticipated opportunity cost in the TFA [28], they weighed the benefits and cons of participating and adjusted their schedule accordingly so that they could attend. The initial enthusiasm to participate in the program and the limited initial hesitation may also be important indicators of self-efficacy, another TFA dimension, and signal that the participants were confident they would attend the intervention.

The attendance rates for this multi-session program were higher compared to other multi-session family interventions in LMICs [44,49] and participants seemed to have experienced a low burden in participation. When we inquired into the facilitators and barriers to program attendance, the content learned in the sessions was the main facilitator mentioned by caregivers and adolescents. They thought that the program benefits were too good to pass and willingly postponed or rescheduled their other commitments. Along similar lines, when they encountered situations that could not be postponed or rescheduled (e.g., sickness), a barrier mentioned by some caregivers, they ensured that another adult family member attended instead so that they would not miss the content. Similarly, adolescent girls willingly rearranged their other commitments (e.g., house chores and Arabic classes) to attend the sessions. In other words, while the experienced opportunity cost [28] was that both caregivers and adolescents had to give up or reschedule other commitments, they willingly did so, which further contextualizes the high attendance rate. The decision to participate and stay engaged in an intervention or program despite anticipated and experienced opportunity costs and burden is a critical indicator of acceptability that could be better incorporated into the TFA.

Other facilitators included the timing of the sessions (weekends) and transportation refund, though mentioned by fewer participants. Costs associated with transportation are widely cited as a barrier to program/ intervention attendance [28,50], especially in low-resource communities [51]. Providing transport refunds was a strategy to minimize families’ attendance at the sessions. However, one should acknowledge that this has cost implications for the implementation and scalability of the intervention. In addition, some adolescents also mentioned the limited availability of public transportation as a barrier that interfered with their timely arrival to the sessions. Hence, structural barriers in low-resource settings need to be considered.

While barriers and facilitators to attendance are not included in the TFA, a better understanding of the contextual factors contributing to attendance may have important implications for assessing intervention acceptability. In fact, Casale et al. [52] underlined the importance of better conceptualizing the social (and political) context in which the interventions are implemented. Some of the facilitators and barriers mentioned in this study reflect the structural and cultural contexts that impact participants’ ability to partake in the program. While perceived barriers have been conceptualized as correlates of acceptability [53], it would be worth considering the value of adding it as a construct to the TFA.

While ethicality did not emerge as a theme in our data, at least some participants likely considered whether the program would be in direct conflict with their value system when enrolling or continuing to attend. It is also important to note that some participants discussed how some aspects of their value systems shifted as a result of the intervention, including their perspectives on girls’ education and expectations in child-caregiver relations.

Caregivers and adolescents found the content and skills provided during the intervention highly relevant to their life circumstances and needs. Perceived content relevance has been cited as an indicator of intervention acceptability in other studies [54,55]. Content relevance is distinct from intervention coherence and perceived effectiveness that are represented in the TFA. While program participants may easily understand the intervention content, they may still find the content irrelevant. Similarly, they may find the content relevant, but the intervention ineffective. Hence, content relevance should be considered as a separate acceptability construct in the TFA.

Relatedly, not only did participants appreciate acquiring knowledge and skills but they also discussed how the intervention made a positive impact on their families. Other studies also identified positive impact as a reason for intervention acceptability [56,57,58,59]. Participants described in detail how their family relations improved with the intervention and they became more “united” as a family. They also discussed how the skills and knowledge acquired in the economic empowerment component allowed them to save, invest in education, and expand business opportunities, among others. Hence, one can assume that the participants were able to understand the intervention and apply its content to their lives, referred to as intervention coherence in TFA. Relatedly, participants also reported the positive impact of the intervention, an aspect of acceptability defined as the experienced perceived effectiveness in the TFA. Considering families’ initial motivations to participate in the intervention were to improve their family relations and learn about finances and saving, one can conclude that the intervention was able to fulfill their anticipated perceived effectiveness.

Overall, our findings suggest that both caregivers and adolescents found the ANZANSI intervention highly acceptable, an important and promising step for this pilot study. Furthermore, the narratives about the perceived effectiveness of the intervention -anticipated and experienced- also point to the high need for this intervention in the communities where the study was implemented, with potential implications for other low-resource communities in sub-Saharan Africa experiencing similar challenges. Finally, our results show that when relevant and acceptable, interventions with multiple sessions can still attain high attendance rates. The next step for this intervention would be to determine its acceptability and effectiveness in a larger sample of participants and schools. Given the barriers around transportation, other potential venues or provision of transport from a primary location to the program site could be considered.

Study results should be interpreted in light of the study limitations. The qualitative interviews were conducted cross-sectionally upon completion of the intervention. Hence, the data do not capture any changes that may occur in families’ perspectives about the intervention over time. Additionally, the TFA was used at the stage of data analysis, rather than at the stage of developing the interview protocol. Relatedly, the interviews did not explicitly explore certain constructs such as ethicality and intervention coherence. In addition, future research should examine the acceptability and potential adoption of the intervention from the perspectives of intervention facilitators and school leadership as these would have implications for intervention sustainability. In addition, further research should be conducted to examine the impact of the intervention as acceptability may not always translate to intervention impact on targeted outcomes.

## 5. Conclusions

Despite these limitations, our study provides important insights into the acceptability of a family combination intervention, ANZANSI Family Program, aiming to reduce the risk of unaccompanied migration of adolescent girls from the Northern region to big cities in Ghana to work as load carriers. Guided by the TFA, our results suggest that both caregivers and adolescent girls found the intervention highly acceptable and that the intervention filled a significant gap for families in the study region. Given that ANZANSI was tested in a pilot study, its acceptability provides a strong ground to further test the acceptability and effectiveness of this preventive intervention in a larger randomized clinical trial in the study area. This is particularly promising as preventive efforts to curtail adolescent girls’ unaccompanied migration for child labor is limited.

## Figures and Tables

**Table 1:** Child demographics.

Variable	Total Sample (N = 20) % (n)
***Age (Mean, SD)***	13.85 (0.37)
***Class Level***	
Junior High School 1	85.0 (17)
Junior High School 2	15.0(3)
***Orphan hood Status***	
Single orphan (Orphan)	20.0 (4)
Non-orphan	80.0 (16)
***Primary Caregiver***	
Biological parent	70.0 (14)
Grandparent	10.0 (2)
Other relatives	20.0 (4)
***Household size (Median, SD)***	
Number of people in HH (min/max = 5–26)	16 (6.89)
Number of children in HH (min/max = 1–15)	6 (3.67)

**Table 2:** Caregiver demographics.

Variable	Total Sample (N = 20) % (n)
***Age (Mean, SD)***	42.85 (13.25)
***Gender***	
Female	85 (17)
Male	15 (3)
***Occupation***	
Informal Employment	75 (15)
Formal Employment	15 (3)
Unemployed	5 (1)
Retired	5 (1)

**Table 3:** Overview of themes and subthemes in relation to the TFA.

Theme	Subtheme	Related TFA Construct	
Motivation to participate in the program	- Opportunity to open bank accounts - Learn about savings, financial literacy, and IGAs - Potential to improve family relations - Focus on girls and their education	- Anticipated affective attitude - Anticipated perceived effectiveness	Self-efficacy
Initial concerns about program participation	- Time commitment - Rumors the program should not be trusted	- Anticipated burden - Anticipated opportunity cost	
Facilitators to program attendance	- Content learned - Rearranging commitments - Finding alternative family adults to attend - Timing of sessions - Transport refund	- Experienced opportunity cost - Experienced burden	
Barriers to program attendance	- Family and other commitments - Transport cost - Limited availability of public transportation	- Experienced opportunity cost - Experienced burden	
Content relevance and perceived impact	- Learning about opening a bank account - Learning about saving - Expanding business - Depositing in the bank - Relating effectively with children - Becoming united as a family - Adjusting expectations from children - Shifting perspectives on girls’ education	- Experienced perceived effectiveness - Intervention coherence	

## Data Availability

The data presented in this study are available on request from the corresponding author. The data are not publicly available due to the fact the qualitative interviews contain sensitive information.

## References

[1] International Labor Organization (ILO) and UNICEF (2021). Child Labor: Global Estimates 2020, Trends and the Road forward. ILO and UNICEF, New York. https://data.unicef.org/resources/child-labour-2020-global-estimates-trends-and-the-road-forward/.

[2] United States Department of Labor 2020 Findings on the Worst Forms of Child Labor: Ghana. https://www.dol.gov/sites/dolgov/files/ILAB/child_labor_reports/tda2020/Ghana.pdf.

[3] United States Department of Labor 2021 Findings on the Worst Forms of Child Labor: Ghana. https://www.dol.gov/sites/dolgov/files/ILAB/child_labor_reports/tda2021/Ghana.pdf.

[4] Anderson K., Apland K., Yarrow E. (2017). Unaccompanied and unprotected: The systemic vulnerability of unaccompanied migrant children in South Africa. The United Nations Convention on the Rights of the Child.

[5] Baah-Ennumh T.Y., Adoma M.O. (2012). The Living Conditions of Female Head Porters in the Kumasi Metropolis, Ghana. J. Soc. Dev. Sci..

[6] Emerson P.M., Souza A.P. (2007). Child Labor, School Attendance, and Intrahousehold Gender Bias in Brazil. World Bank Econ. Rev..

[7] International Labor Organization (2013). Children on the Move. Geneva: Switzerland. http://publications.iom.int/bookstore/free/Children_on_the_Move_15May.pdf.

[8] Heady C. (2003). The Effect of Child Labor on Learning Achievement. World Dev..

[9] Khanam R. (2008). Child labour and school attendance: Evidence from Bangladesh. Int. J. Soc. Econ..

[10] Shabaya J., Konadu-Agyemang K. (2004). Unequal access, unequal participation: Some spatial and socio-economic dimensions of the gender gap in education in Africa with special reference to Ghana, Zimbabwe and Kenya. Comp. A J. Comp. Int. Educ..

[11] Ertürk Y., Dayıoğlu M. (2004). Gender, Education and Child Labour in Turkey.

[12] Kwankye S.O. (2012). Independent North-South Child Migration as a Parental Investment in Northern Ghana. Popul. Space Place.

[13] Blunch N., Verner D. (2000). Revisiting the Link between Poverty and Child Labor: The GHANAIAN Experience.

[14] Awumbila M. (2006). Gender equality and poverty in Ghana: Implications for poverty reduction strategies. GeoJournal.

[15] Boateng J.K., Adomako-Ampofo A., Flanagan C.C., Gallay L., Yakah J., Texas A. Gender socialization of pre-teen youths in Ghana: Alternative approaches for extension. Proceedings of the 22nd Annual Conference of the Association of International Agricultural & Extension Education (AIAEE).

[16] Kwankye S.O., Anarfi J.K., Tagoe C.A., Castaldo A. (2007). Coping strategies of independent child migrants from Northern Ghana to Southern cities. Development Research Centre on Migration, Globalization and Poverty Working Paper Series.

[17] Awumbila M., Ardayfio-Schandorf E. (2008). Gendered poverty, migration and livelihood strategies of female porters in Accra, Ghana. Nor. Geogr. Tidsskr. Nor. J. Geogr..

[18] Yeboah M.A., Appiah-Yeboah K. (2009). An examination of the cultural and socio-economic profiles of porters in Accra, Ghana. Nord. J. Afr. Stud..

[19] Amerikaner M., Monks G., Wolfe P., Thomas S. (1994). Family Interaction and Individual Psychological Health. J. Couns. Dev..

[20] Miller K.S., Forehand R., Kotchick B.A. (1999). Adolescent Sexual Behavior in Two Ethnic Minority Samples: The Role of Family Variables. J. Marriage Fam..

[21] Han C.-K., Ssewamala F.M., Wang J.S.-H. (2013). Family economic empowerment and mental health among AIDS-affected children living in AIDS-impacted communities: Evidence from a randomised evaluation in southwestern Uganda. J. Epidemiology Community Health.

[22] McKay M.M., Gonzales J.J., Stone S., Ryland D., Kohner K. (1995). Multiple Family Therapy Groups: A responsive intervention model for inner city families. Soc. Work Groups.

[23] Olsson J., Höjer S., Nyström L., Emmelin M. (2017). Orphanhood and mistreatment drive children to leave home—A study from early AIDS-affected Kagera region, Tanzania. Int. Soc. Work.

[24] Sorsa S., Abera A. (2006). A Study on child labor in three major towns of southern Ethiopia. Ethiop. J. Health Dev..

[25] Buchmann C. (2000). Family Structure, Parental Perceptions, and Child Labor in Kenya: What Factors Determine Who is Enrolled in School?. Soc. Forces.

[26] Bermudez L.G., Bahar O.S., Dako-Gyeke M., Boateng A., Ibrahim A., Ssewamala F.M., McKay M. (2018). Understanding female migrant child labor within a cumulative risk framework: The case for combined interventions in Ghana. Int. Soc. Work.

[27] Somefun O.D., Casale M., Ronnie G.H., Desmond C., Cluver L., Sherr L. (2021). Decade of research into the acceptability of interventions aimed at improving adolescent and youth health and social outcomes in Africa: A systematic review and evidence map. BMJ Open.

[28] Sekhon M., Cartwright M., Francis J.J. (2017). Acceptability of healthcare interventions: An overview of reviews and development of a theoretical framework. BMC Health Serv. Res..

[29] Yeager D.S., Dahl R.E., Dweck C.S. (2018). Why Interventions to Influence Adolescent Behavior Often Fail but Could Succeed. Perspect. Psychol. Sci..

[30] Gaskins S. (1996). How Mayan parental theories come into play. Parents’ Cultural Belief Systems: Their Origins, Expressions, and Consequences.

[31] Sherraden M., Gilbert N. (1996). Assets and the Poor: New American Welfare Policy.

[32] Goodnow J.J. (1996). From household practices to parent’s ideas about work and interpersonal relationships. Parents’ Cultural Belief Systems: Their Origins, Expressions, and Consequences.

[33] Bahar O.S., Ssewamala F.M., Ibrahim A., Boateng A., Nabunya P., Neilands T.B., Asampong E., McKay M.M. (2020). Anzansi family program: A study protocol for a combination intervention addressing developmental and health outcomes for adolescent girls at risk of unaccompanied migration. Pilot Feasibility Stud..

[34] Byansi W., Ssewamala F.M., Neilands T.B., Bahar O.S., Nabunya P., Namuwonge F., McKay M.M. (2022). The Short-Term Impact of a Combination Intervention on Depressive Symptoms among School-Going Adolescent Girls in Southwestern Uganda: The Suubi4Her Cluster Randomized Trial. J. Adolesc. Health.

[35] Wang J.S.H., Ssewamala F.M., Neilands T.B., Bermudez L.G., Garfinkel I., Waldfogel J., Brooks-Gunn J., You J. (2018). Effects of financial incentives on saving outcomes and material well-being: Evidence from a randomized controlled trial in Uganda. J. Policy Anal. Manag..

[36] Ssewamala F.M., Karimli L., Torsten N., Wang J.S.-H., Han C.-K., Ilic V., Nabunya P. (2016). Applying a Family-Level Economic Strengthening Intervention to Improve Education and Health-Related Outcomes of School-Going AIDS-Orphaned Children: Lessons from a Randomized Experiment in Southern Uganda. Prev. Sci..

[37] Dennison S.T. (2005). A Multiple Family Group Therapy Program for at Risk Adolescents and Their Families.

[38] Brathwaite R., Ssewamala F.M., Bahar O.S., McKay M.M., Neilands T.B., Namatovu P., Kiyingi J., Zmachinski L., Nabayinda J., Huang K. (2022). The longitudinal impact of an evidence-based multiple family group intervention (Amaka Amasanyufu) on oppositional defiant disorder and impaired functioning among children in Uganda: Analysis of a cluster randomized trial from the SMART Africa-Uganda scale-up study (2016–2022). J. Child Psychol. Psychiatry.

[39] Bahar O.S., Byansi W., Kivumbi A., Namatovu P., Kiyingi J., Ssewamala F.M., McKay M.M., Nyoni T. (2020). From “4Rs and 2Ss” to “Amaka Amasanyufu” (Happy Families): Adapting a U.S.-based Evidence-Based Intervention to the Uganda Context. Fam. Process.

[40] Asampong E., Ibrahim A., Sensoy-Bahar O., Kumbelim K., Yaro P.B., McKay M.M., Ssewamala F.M. (2021). Adaptation and Implementation of the Multiple-Family Group Intervention in Ghana. Psychiatr. Serv..

[41] Lincoln Y.S., Guba E.G. (1985). Naturalistic Inquiry.

[42] Padgett D.K. (2008). Qualitative Methods in Social Work Research.

[43] Geng E.H., Nash D., Kambugu A., Zhang Y., Braitstein P., Christopoulos K.A., Muyindike W., Bwana M.B., Yiannoutsos C.T., Petersen M.L. (2010). Retention in Care among HIV-Infected Patients in Resource-Limited Settings: Emerging Insights and New Directions. Curr. HIV/AIDS Rep..

[44] Shenderovich Y., Eisner M., Cluver L., Doubt J., Berezin M., Majokweni S., Murray A.L. (2018). What Affects Attendance and Engagement in a Parenting Program in South Africa?. Prev. Sci..

[45] Yeasmin F., Winch P.J., Hwang S.T., Leontsini E., Jahir T., Das J.B., Amin M.R., Hossain K., Huda T.M.N., Akter F. (2021). Exploration of Attendance, Active Participation, and Behavior Change in a Group-Based Responsive Stimulation, Maternal and Child Health, and Nutrition Intervention. Am. J. Trop. Med. Hyg..

[46] Contractor L.F.M., Celedonia K.L., Cruz M., Douaihy A., Kogan J.N., Marin R., Stein B.D. (2010). Mental Health Services for Children of Substance Abusing Parents: Voices from the Community. Community Ment. Health J..

[47] Field S., Abrahams Z., Honikman S. (2020). Adolescent mothers: A qualitative study on barriers and facilitators to mental health in a low-resource setting in Cape Town, South Africa. Afr. J. Prim. Health Care Fam. Med..

[48] Hodge L.M., Turner K.M.T. (2016). Sustained Implementation of Evidence-based Programs in Disadvantaged Communities: A Conceptual Framework of Supporting Factors. Am. J. Community Psychol..

[49] Annan J., Sim A., Puffer E.S., Salhi C., Betancourt T.S. (2017). Improving Mental Health Outcomes of Burmese Migrant and Displaced Children in Thailand: A Community-Based Randomized Controlled Trial of a Parenting and Family Skills Intervention. Prev. Sci..

[50] Murray L.K., Skavenski S., Michalopoulos L.M., Bolton P.A., Bass J.K., Familiar I., Imasiku M., Cohen J. (2014). Counselor and Participant Perspectives of Trauma-Focused Cognitive Behavioral Therapy for Children in Zambia: A Qualitative Study. J. Clin. Child Adolesc. Psychol..

[51] Mathews C., Eggers S.M., De Vries P.J., Mason-Jones A.J., Townsend L., Aarø L.E., De Vries H. (2015). Reaching the hard to reach: Longitudinal investigation of adolescents’ attendance at an after-school sexual and reproductive health programme in Western Cape, South Africa. BMC Public Health.

[52] Casale M., Yates R., Gittings L., Ronnie G.H., Somefun O., Desmond C. (2022). Consolidate, conceptualize, contextualise: Key learnings for future intervention acceptability research with young people in Africa. Psychol. Health Med..

[53] Ortblad K.F., Sekhon M., Wang L., Roth S., van der Straten A., Simoni J.M., Velloza J. (2022). Acceptability Assessment in HIV Prevention and Treatment Intervention and Service Delivery Research: A Systematic Review and Qualitative Analysis. AIDS Behav..

[54] Patel V., Chowdhary N., Rahman A., Verdeli H. (2011). Improving access to psychological treatments: Lessons from developing countries. Behav. Res. Ther..

[55] Parker L., Maman S., Pettifor A., Chalachala J.L., Edmonds A., Golin C.E., Moracco K., Behets F. (2013). SYMPA Study Team Feasibility Analysis of an Evidence-Based Positive Prevention Intervention for Youth Living With HIV/AIDS in Kinshasa, Democratic Republic of the Congo. AIDS Educ. Prev..

[56] Banda E., Svanemyr J., Sandøy I.F., Goicolea I., Zulu J.M. (2019). Acceptability of an economic support component to reduce early pregnancy and school dropout in Zambia: A qualitative case study. Glob. Health Action.

[57] Chirwa-Kambole E., Svanemyr J., Sandøy I., Hangoma P., Zulu J.M. (2020). Acceptability of youth clubs focusing on comprehensive sexual and reproductive health education in rural Zambian schools: A case of Central Province. BMC Health Serv. Res..

[58] Kansiime C., Hytti L., Nalugya R., Nakuya K., Namirembe P., Nakalema S., Neema S., Tanton C., Alezuyo C., Musoke S.N. (2020). Menstrual health intervention and school attendance in Uganda (MENISCUS-2): A pilot intervention study. BMJ Open.

[59] Sabben G., Mudhune V., Ondeng’E K., Odero I., Ndivo R., Akelo V., Winskell K. (2019). A Smartphone Game to Prevent HIV Among Young Africans (Tumaini): Assessing Intervention and Study Acceptability Among Adolescents and Their Parents in a Randomized Controlled Trial. JMIR mHealth uHealth.

