# Correction: Sensoy Bahar et al. “*ANZANSI Program Taught Me Many Things in Life*”: Families’ Experiences with a Combination Intervention to Prevent Adolescent Girls’ Unaccompanied Migration for Labor. *Int. J. Environ. Res. Public Health* 2022, *19*, 13168

**DOI:** 10.3390/ijerph20166590

**Published:** 2023-08-17

**Authors:** Ozge Sensoy Bahar, Alice Boateng, Portia B. Nartey, Abdallah Ibrahim, Kingsley Kumbelim, Proscovia Nabunya, Fred M. Ssewamala, Mary M. McKay

**Affiliations:** 1 Brown School, Washington University in St. Louis, Campus Box 1196, One Brookings Drive, St. Louis, MO 63130, USA; 2 Department of Social Work, University of Ghana, Accra P.O. Box LG419, Ghana; 3 School of Public Health, University of Ghana, Accra P.O. Box LG419, Ghana; 4 BasicNeeds Ghana, Tamale P.O. Box TL1140, Ghana; 5 Vice Provost Office, Washington University in St. Louis, One Brookings Drive, St. Louis, MO 63130, USA

## Text Correction

There was an error in the original publication [1]. The second paragraph in Section 3.3 of the results section was misplaced. A correction has been made to the Results section, Section 3.3, and the second paragraph was moved to the end of Section 3.2.

Adolescent girls also mentioned the content of the sessions as one of the reasons that motivated them to come to the sessions. For instance, when asked about what made it easy for her to attend the sessions, Ayat said, “They were teaching us how you will save money, how to fill the deposit slip, and what you supposed to do when you go to a bank…. They were educating us on how a child should live.” She added that she experienced no challenges in attending the sessions. Similarly, Patricia, who never missed a session, shared, “We never missed a session because we know it is something that is going to help us in relation to our family and education”.

A correction was also made to the data collection period in the Methods section, Section 2.3, in the first paragraph.

Face-to-face semi-structured interviews were conducted with adolescent girls and their caregivers separately following the completion of the ANZANSI intervention, between December 2021 and January 2022.

The authors apologize for any inconvenience caused and state that the scientific conclusions are unaffected. The original publication has also been updated.
